# Fabrication and characterization of 3D-printed composite scaffolds of coral-derived hydroxyapatite nanoparticles/polycaprolactone/gelatin carrying doxorubicin for bone tissue engineering

**DOI:** 10.1007/s10856-024-06779-x

**Published:** 2024-01-29

**Authors:** Fatima Kadi, Ghasem Dini, S. Ali Poursamar, Fatemeh Ejeian

**Affiliations:** 1 Department of Nanotechnology, Faculty of Chemistry, University of Isfahan, Isfahan, 81746-73441 Iran; 2 Department of Biomaterials, Nanotechnology, and Tissue Engineering, School of Advanced Technologies in Medicine, Isfahan University of Medical Sciences, Isfahan, Iran; 3 Department of Animal Biotechnology, Cell Science Research Center, Royan Institute for Biotechnology, ACECR, Isfahan, 81593-58686 Iran

## Abstract

In this study, nanocomposite scaffolds of hydroxyapatite (HA)/polycaprolactone (PCL)/gelatin (Gel) with varying amounts of HA (42–52 wt. %), PCL (42–52 wt. %), and Gel (6 wt. %) were 3D printed. Subsequently, a scaffold with optimal mechanical properties was utilized as a carrier for doxorubicin (DOX) in the treatment of bone cancer. For this purpose, HA nanoparticles were first synthesized by the hydrothermal conversion of Acropora coral and characterized by using different techniques. Also, a compression test was performed to investigate the mechanical properties of the fabricated scaffolds. The mineralization of the optimal scaffold was determined by immersing it in simulated body fluid (SBF) solution for 28 days, and the biocompatibility was investigated by seeding MG-63 osteoblast-like cells on it after 1–7 days. The obtained results showed that the average size of the synthesized HA particles was about 80 nm. The compressive modulus and strength of the scaffold with 47 wt. % HA was reported to be 0.29 GPa and 9.9 MPa, respectively, which was in the range of trabecular bones. In addition, the scaffold surface was entirely coated with an apatite layer after 28 days of soaking in SBF. Also, the efficiency and loading percentage of DOX were obtained as 30.8 and 1.6%, respectively. The drug release behavior was stable for 14 days. Cytotoxicity and adhesion evaluations showed that the fabricated scaffold had no negative effects on the viability of MG-63 cells and led to their proliferation during the investigated period. From these results, it can be concluded that the HA/PCL/Gel scaffold prepared in this study, in addition to its drug release capability, has good bioactivity, mechanical properties, and biocompatibility, and can be considered a suitable option for bone tumor treatment.

**Graphical Abstract:**

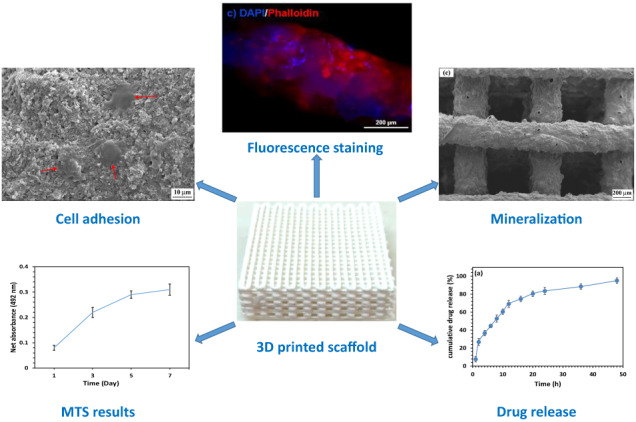

## Introduction

Bone tissue engineering (BTE) has emerged as a promising alternative to traditional bone grafts, driven by its potential for an endless supply and the absence of disease transmission. Despite these advantages, BTE procedures have not progressed into clinical practice, primarily due to various constraints. The aim is to harness the regenerative capabilities of local or implanted stem/progenitor cell populations by integrating biodegradable and osteoconductive three-dimensional (3D) scaffolds with controlled delivery of osteoinductive molecules [1, 2].

Addressing large and serious bone defects and promoting bone regeneration stands as a crucial concern for orthopedic surgeons, as highlighted by Alidadi et al. [3]. In response, numerous researchers have undertaken extensive efforts to identify methodologies with minimal side effects [4, 5]. The utilization of 3D-printing technology in BTE has proven effective, thanks to its rapid, precise, and controlled production process. While conventional bioceramic scaffolds are commonly employed in BTE, the development of bioceramic scaffolds with a hierarchical structure, comprising macro-, micro-, and nanomaterials, has gained prominence. This innovation aims to provide a 3D environment conducive to cell adhesion and proliferation [6–9].

Extensive research has focused on bioceramics for numerous years, driven by their similarity to the inorganic composition of bone. Bioceramics possess notable characteristics such as high stiffness, hydrophobicity, bioactivity, biocompatibility, osteoconductivity, and potential osteoinductivity, all contributing to their ability to promote bone regeneration by modifying in vivo conditions. Ceramics offer a significant advantage over other implant materials due to their varied biocompatibility, some are inert under biological conditions, while others elicit a regulated response in the body. Bioactive ceramics, including hydroxyapatite (HA), glass ceramics, and bioactive glasses, interact with biological fluids through cellular activity, bridging the gap between hard and soft tissues [10, 11]. Frequently employed as metal-support coatings, HA, with its chemical composition (Ca10(PO4)6(OH)2) identical to the main constituents of bone, has been extensively studied in BTE. It has demonstrated beneficial effects on osteoblast adhesion and proliferation, being a key component of the mineral phases in teeth and bones. Notably osteoconductive, biocompatible, and non-toxic, HA, along with other calcium phosphate compounds, holds a special place in biomedical and dental materials [12–14].

The increasing demand for novel, intricate, and multifunctional materials has brought attention to natural composite materials that underwent substantial modification through extended evolution, selection pressures, and adaptation processes. Among these, marine biological materials stand out as vital sources of inspiration for biomimicry and raw materials with applications spanning technology and biomedicine [15]. Studies on natural engineering structures, or biomimicry as defined by Vincent et al. [16], have offered diverse methods for creating distinctive scaffolds applicable in regenerative medicine, showing promise to significantly enhance conventional human-made biomaterials. Corals, with their osteoconductivity, biocompatibility, and favorable dissolution qualities, emerge as effective candidates for scaffold development [17]. Utilizing coral as the raw material results in a porous implant structure, facilitating the proliferation and invasion of hard and soft tissues. This structural characteristic fosters the formation of robust mechanical and chemical bonds. Furthermore, leveraging coral as the starting material offers the advantage of shape customization before conversion into HA [18].

Only a select few coral genera, notably Porites, Goniopora, and Acropora, exhibit morphological and structural features almost identical to bone. These corals possess the potential to serve as temporary bone replacements due to their striking resemblance to bone structure. Characterized by extensive networks of channels and pores, these corals display a geometrical arrangement of connected spaces in two main directions, mirroring the pore configurations of decellularized bone. This structural similarity facilitates the permeation of new blood vessels and, ultimately, the development of endogenous bone. In particular, Acropora corals stand out for their specialization in resisting strong mechanical loads, thanks to their compact structure and low porosity. Additionally, their irregular yet well-organized pores contribute to enhanced permeability [19, 20]. Various HA-based degradable polymer composites, such as HA/collagen, HA/gelatin (Gel), and HA/polycaprolactone (PCL), have been employed in bone tissue engineering, exhibiting favorable mechanical properties and remarkable bioproperties. For instance, HA/collagen composites have shown improvements in the adhesion, proliferation, and differentiation of seeded stem cells. In a study by Hamlekhan et al. [21], PCL/HA/Gel composite scaffolds were investigated to enhance mechanical effectiveness, leading to increased stress, stiffness, and compressive modulus. The incorporation of HA in the HA/Gel composite resulted in heightened compressive modulus and toughness, reaching approximately 0.18 GPa, remarkably similar to that of natural sponge bone. Similarly, in the HA/PCL scaffold, an increase in HA content from 0 to 30 wt. % correlated with a rise in compressive modulus from 0.3 to 0.5 GPa, marking a 2.4-fold increase compared to PCL alone [22].

The slow degradation rate, poor mechanical strength, and low fracture toughness of pure HA can hinder complete bone regeneration and increase the risk of infection. To address these limitations, composite materials with desirable features, including porosity, mechanical strength, thermal properties, regulated degradation rates, and the incorporation of bioactive substances, are essential for enhanced repair and regeneration in bone tissue engineering. In addition, porous HA/PCL scaffolds have demonstrated a more efficient promotion of osteoblast proliferation and viability compared to pure PCL scaffolds. Gómez-Lizárraga et al. [23] conducted a study comparing 3D-printed scaffolds made of pure PCL, PCL/synthetic-HA, and PCL/bio-HA derived from bovine bones. The findings revealed that PCL/bio-HA scaffolds exhibit enhanced bioactivity over pure PCL, fostering improved cell adhesion, activation, and proliferation. These composite materials capitalize on the advantages offered by a variety of biodegradable materials, finding extensive applications in BTE. Instead of relying solely on either natural polymers (e.g., collagen, gelatin, alginate, hyaluronic acid, and chitosan) or synthetic polymers (e.g., poly(lactic-co-glycolic acid) (PLGA), polylactic acid (PLA), and PCL), and bioceramics like HA, composite forms have gained widespread use in BTE [24–26].

This study aimed to evaluate the synthesis of HA nanoparticles derived from a biomimetic source. Subsequently, the effectiveness of HA/PCL/Gel scaffolding, optimized for HA percentage, was investigated in terms of bioactivity, biodegradability, viability, and the release profile of doxorubicin (DOX).

In previous studies, researchers have explored the utilization of HA, PCL, and Gel in scaffold fabrication, acknowledging their merits in bone regeneration and drug delivery systems. However, a comprehensive understanding of how varying compositions of these components impact the mechanical properties and drug-release capabilities of 3D-printed nanocomposite scaffolds remains an area requiring further exploration. Furthermore, while the bioactivity of scaffolds in simulated body fluid (SBF) has been studied, the specific implications of mineralization on the scaffold’s potential for bone tumor treatment have yet to be fully elucidated.

This study aims to build upon the foundation laid by previous research by systematically investigating the influence of different ratios of HA, PCL, and Gel on the mechanical properties and drug release profiles of 3D-printed nanocomposite scaffolds. Additionally, we delve into the mineralization process of the optimal scaffold in SBF, unraveling its implications for bioactivity and its potential in the context of bone tumor treatment. Through these endeavors, we strive to contribute valuable insights to the evolving landscape of bone tissue engineering, identifying pathways for innovation and addressing unmet needs in the field.

## Materials and Methods

### Materials

Acropora coral was sourced from Kish Island, Iran. PCL with a molecular weight of 80 kDa, phosphoric acid, ammonia solution, and Gel powder were procured from Sigma-Aldrich, Germany. Phosphate-buffered saline (PBS) and simulated body fluid (SBF) were obtained from Topal Advance Materials, Iran.

### Synthesis of coral-derived HA

The method proposed by Roy and Linnehan [27] was employed in this study to convert coral powder into HA through a hydrothermal process. Initially, coral powder was prepared by mechanically milling crushed and washed corals for 5 h in a planetary ball mill, utilizing zirconia (ZrO_2_) balls and cups with a ball-to-powder ratio of 30:1. This milling process was repeated until the desired amount of powder was obtained. Subsequently, 2 g of milled coral powder was combined with 1.6 mL of H_3_PO_4_ and distilled water (100 mL) at a Ca/P ratio of 1.67. The pH was adjusted to 7 by introducing an NH_4_OH solution. After thorough stirring, the mixture was tightly sealed in a 200 mL Teflon-lined stainless-steel autoclave and subjected to an oven at 180 °C for 46 h. The resulting product was filtered, washed with distilled water, and then dried in an oven at 80 °C for one day.

### Fabrication of 3D scaffolds

Three ink compositions with varying ratios of the constituent materials were prepared (see Table 1). To mitigate the risk of agglomeration and potential nozzle clogging during printing, the synthesized powder underwent a sieving process using a 150-mesh-size sieve. Subsequently, PCL was dissolved in chloroform within a sealed container at 40 °C for 2 h. The dissolved PCL was then blended with Gel and the sieved HA. The Gel content remained constant across all three ink compositions. Additionally, a pure PCL scaffold was manufactured for comparative purposes.

**Table 1:** Composition of three different inks used for scaffold fabrication

Material	Ink 1	Ink 2	Ink 3
HA (wt. %)	42	47	52
PCL (wt. %)	52	47	42
Gel (wt. %)	6	6	6

A NIKA 3D printer from Adli Regeneration Medicine Company in Isfahan, Iran, was employed for the printing process, with the Repetier Host Software used to control the printer. The printing geometry, a 2 × 2 cm^2^ cubic-shaped block with a strut spacing of 500 μm, was designed using the Solidworks program ver. 2017 and printed through the Repetier software interface ver. 2.1.6. The Slic3r slicing profile was applied to convert the STL file into G-code, with a designated layer height of 300 μm. The porous 3D constructs were printed layer-by-layer, involving the continuous extrusion of the composite ink for up to 20 layers. The printing ink, a composite of PCL, Gel, and HA, was extruded through a 22 G stainless steel nozzle with an internal diameter of 410 μm.

Printing configurations varied among scaffolds depending on the ink composition. For ink 2, where PCL and HA percentages were equal, the extrusion rate was set at 0.01 mm/s, and the line deposition rate was 2 mm/s. In the case of ink 3, where PCL content was lower than HA, potentially causing agglomeration, the extrusion rate was increased to 0.013 mm/s. Additionally, to address quick ink setting due to low PCL, resulting in cracks, the line deposition rate was increased to 6 mm/s. For ink 1, characterized by stickiness and lower viscosity due to a reduced HA amount, the extrusion rate was decreased to 0.009 mm/s, and the line deposition rate was reset to 2 mm/s.

### Characterization

Different techniques were employed to characterize the raw coral, synthesized HA powder, and fabricated scaffolds. Structural analysis of milled Acropora coral and the synthesized HA powder was performed using X-ray diffraction analysis (XRD) on a D8 Advance Bruker instrument with a wavelength of 1.54 Å. The chemical compositions of the raw coral and synthesized HA were determined through X-ray fluorescence spectrometry (XRF) using a Bruker S4 Pioneer instrument. Morphological investigations of the as-received coral and synthesized HA were conducted using field-emission scanning electron microscopy (SEM) on a ZEISS SIGMA VP-500 instrument. SEM was also employed to characterize the apatite layer formed and cell adhesion on the surface of the fabricated scaffold after biological tests. The specific surface area, pore volume, and average pore diameter of the synthesized HA powder were determined through the Brunauer-Emmett-Teller (BET) method, involving the adsorption/desorption of N_2_ gas at liquid nitrogen temperature (~77 K) using a Series BEL SORP mini II. Mechanical compression tests were performed using a 2 T SANTAM testing machine. Three specimens of approximately 5 × 5 × 5 mm^3^ were tested for each scaffold group. The compressive modulus for each sample was determined by linearly fitting the elastic part of the stress-strain curve.

### Biodegradability

To assess the in vitro behavior of the scaffolds, a degradation test was employed. Initially, the samples were weighed (W_0_) before immersion in PBS. Following incubation in PBS at 37 °C for 7, 14, 21, and 28 days, the samples were retrieved and wiped, and the pH of PBS was measured every 24 h. Subsequently, the samples were washed with PBS and placed in a vacuum oven at 37 °C for 12 h. Their weights were then measured (W_t_), and Eq. 1 was applied to calculate the weight loss. Additionally, concentrations of calcium and phosphorus ions in the PBS solution were determined using inductively coupled plasma atomic emission spectroscopy (ICP-OES) on an Analytik Jena PQ 9000 instrument.

1 $${\rm{Degradation}}\,( \% )=\frac{({W}_{0}-{W}_{t})}{{W}_{0}}\times 100$$

### Bioactivity

To evaluate the in vitro mineralization activity of the scaffolds, they were immersed in SBF. Samples of approximately 5 × 5 × 3 mm^3^ were submerged in 10 mL of SBF (the mass-to-volume ratio of about 20 mg/mL) for 28 days at 37 °C. Subsequently, SEM was employed to examine the formation of an apatite layer on the scaffold surface. In order to assess the bioactivity mechanism, the pH of SBF in the presence of the scaffold samples was measured.

### Drug loading and releasing

A serially diluted solution of the drug was prepared from DOX stock (2 mg/mL, Iranian Red Crescent) with concentrations ranging from 10 to 100 µg/mL. The UV absorption of each solution was measured at 480 nm using a Cary 60 UV-Vis spectrophotometer, and a calibration curve was constructed through the linear regression method.

For the preparation of the sample for drug loading, the scaffold was cut into small disks (120 mg) and subjected to sterilization under UV light for 1 h. Subsequently, a 4 mL tube containing the sterile scaffold received 3 mL of DOX solution, and the mixture was stored for 24 h in a biosafety cabinet. Loading capacity (LC) and entrapment efficiency (EE) were calculated using Eqs. 2 and 3, respectively.

2 $${\rm{LC}}( \% )=\frac{Mass\,of\,drug\,in\,scaf\,fold}{Total\,mass\,of\,used\,drug}\times 100$$

3 $$EE( \% )=\frac{Mass\,of\,drug\,in\,scaf\,fold}{Total\,Mass\,of\,scaf\,fold}\times 100$$

For the in vitro release test, the 3D-printed scaffold loaded with the drug was immersed in 4 mL of PBS solution at 37 °C. The DOX release medium was periodically replaced with fresh PBS at selected time intervals. Subsequently, the UV absorption of each medium was measured using a UV-Vis spectrophotometer at 480 nm. The cumulative concentration of DOX released from the scaffold was then plotted against time.

### In vitro biological assays

#### Cell culture

The MG-63 cell line obtained from the Royan Institute, Iran, was employed in this study. To cultivate the cell line, Dulbecco’s modified Eagle’s medium, comprising 10% fetal bovine serum (FBS), 1% Glutamax, 1% penicillin/streptomycin antibiotic, and 1% essential amino acids, served as the culture medium. Before cell seeding, the scaffolds underwent sterilization in 70% ethanol for 1 h, followed by 30 min of UV irradiation on each side. MG-63 cells were then seeded onto the scaffolds with a density of 20,000 cells/ml for subsequent experiments.

#### Cell adhesion

To assess the scaffolds’ capability for supporting cell attachment, MG-63 cells were cultured on the samples for 24 h. Subsequently, the cells were fixed with 2.5% glutaraldehyde for 2 h at room temperature and dehydrated using increasing concentrations of ethanol (30, 70, and 100%) for 10 min each. The prepared samples were then gold-sputtered and imaged using SEM.

#### Fluorescent staining

To visualize the cell cytoskeleton architecture, the nucleus and actin filaments were stained with 4,6-diamidino-2-phenylindole (DAPI, blue) and TRITC-labeled phalloidin (red), respectively. Following 24 h of scaffold culture, samples were fixed with 4% paraformaldehyde, permeabilized with 0.2% Triton X-100, and subjected to staining with phalloidin and DAPI. Subsequently, imaging was conducted using an Olympus BX51 fluorescence microscope.

#### Cell activity

To assess the metabolic activity of cells cultured on the scaffold, the MTS assay was conducted on days 1, 3, 5, and 7. Before cell seeding, the 96-well plate was coated with polyhydroxy ethyl methacrylate (p-HEMA) to prevent cell adhesion to the plate’s bottom. Following the manufacturer’s instructions, MTS/PMS solution (Promega, WI, USA) was substituted with the culture medium at each time point, and formazan production was measured after 3.5 h using a microplate reader (Fluostar Optima, BMG Lab Technologies, Germany) at 492 nm. The obtained absorption values were normalized to the absorption value of the control sample (containing culture medium + MTS without cells), and the resulting number was reported as the net absorption value.

### Statistical analysis

All quantitative outcomes were presented as the mean (*n* = 3) accompanied by the standard deviation (SD).

## Results

### Characterization of Acropora coral

Initially, XRF analysis was employed to determine the elemental composition of the as-received Acropora corals, and the obtained results are presented in Table 2. A comparison with the findings of Guillemin et al. [28] confirmed that the coral exoskeleton primarily consisted of calcium carbonate (CaCO_3_) with approximately 3 wt. % of other trace elements. After ball-milling, a trace of ZrO_2_ (~0.8 wt. %), originating from the milling balls and cups, was observed in the chemical composition of the coral powder. However, it should be considered that the milling material, ZrO_2_, is so hard that no contamination should occur.

**Table 2:** Semi-quantitative XRF results of as-received Acropora coral after ball milling and synthesized powder via hydrothermal method

As-received coral	CaO	ZrO_2_	MgO	SiO_2_	SO_3_	Na_2_O	Cl	L.O.I
Concentration (wt. %)	52.1	0.8	0.8	0.6	0.6	0.5	0.1	44.5
Synthesized powder	Ca	P	Zr	Mg	Si	S	Na	Ca/P (mol./mol.)
Concentration (wt. %)	36.4	17.1	0.7	0.2	0.1	< 0.1	< 0.1	1.65

**Fig. 1:**
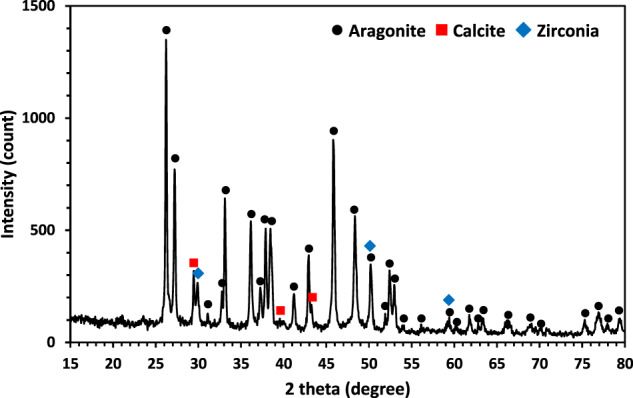
XRD pattern of as-received coral after ball milling

**Fig. 2:**
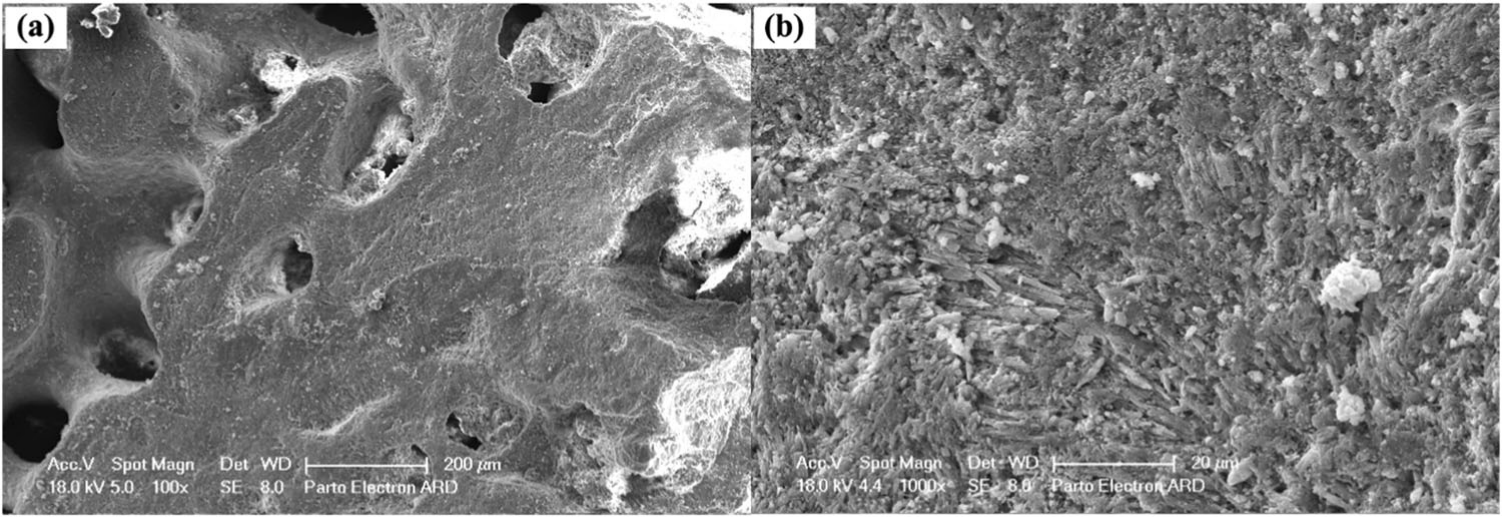
SEM micrographs of as-received coral at two magnifications

XRD analysis was employed to determine the phase structures of the raw corals. Figure 1 illustrates the XRD pattern of the milled coral. The predominant phase in the as-received coral powder is aragonite, a high-pressure stable phase of CaCO_3_, as observed in similar studies by Hansel et al. [29]. Additionally, a minor phase identified as ZrO_2_, resulting from the ball-milling process, was present in the milled coral powder. Calcite peaks, indicative of a stable phase of CaCO_3_ at atmospheric pressure, were also observed in the unconverted coral powder. Notably, the element Ca, as reported by Demers et al. [19], plays a role in bone regeneration when corals are implanted in vivo. SEM analysis was conducted to study the morphology of the raw coral pieces. Figure 2, derived from SEM examination of the unconverted coral, reveals a microporous surface with pore sizes ranging from 5 to 50 μm. According to Wu et al. [30], Acropora exhibits numerous irregularly shaped but similarly oriented pores, featuring the largest pore diameter and highest permeability. This characteristic facilitates fluid transport in vitro, contributing to the success of these constructs in tissue engineering applications.

### Characterization of synthesized HA powder

**Fig. 3:**
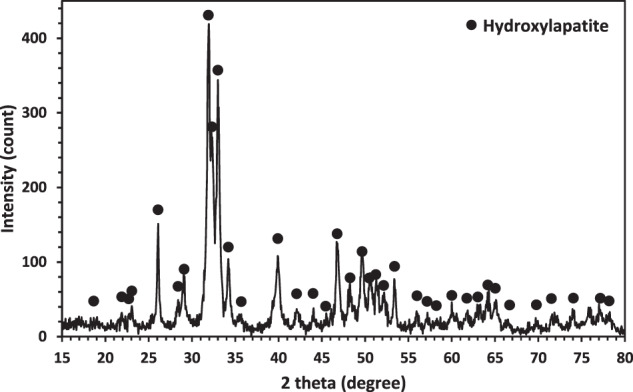
XRD pattern of synthesized powder via hydrothermal method

The results of the XRF analysis for the powder synthesized using the hydrothermal method are presented in Table 2. The Ca/P molar ratio, determined by XRF, closely matched the theoretical value of HA [31], with a calculated value of 1.65. Ca and P constituted 95 wt. % of the total mineral content of the natural HA. Following the hydrothermal process, XRD analysis identified the product as a single-phase highly crystalline HA with sharp peaks, as illustrated in Fig. 3. Notably, no peaks corresponding to CaCO_3_ or CaO were observed, indicating complete conversion. These findings align with a study conducted by Sivakumar et al. [32] on hard coral converted to HA using a hydrothermal process.

**Fig. 4:**
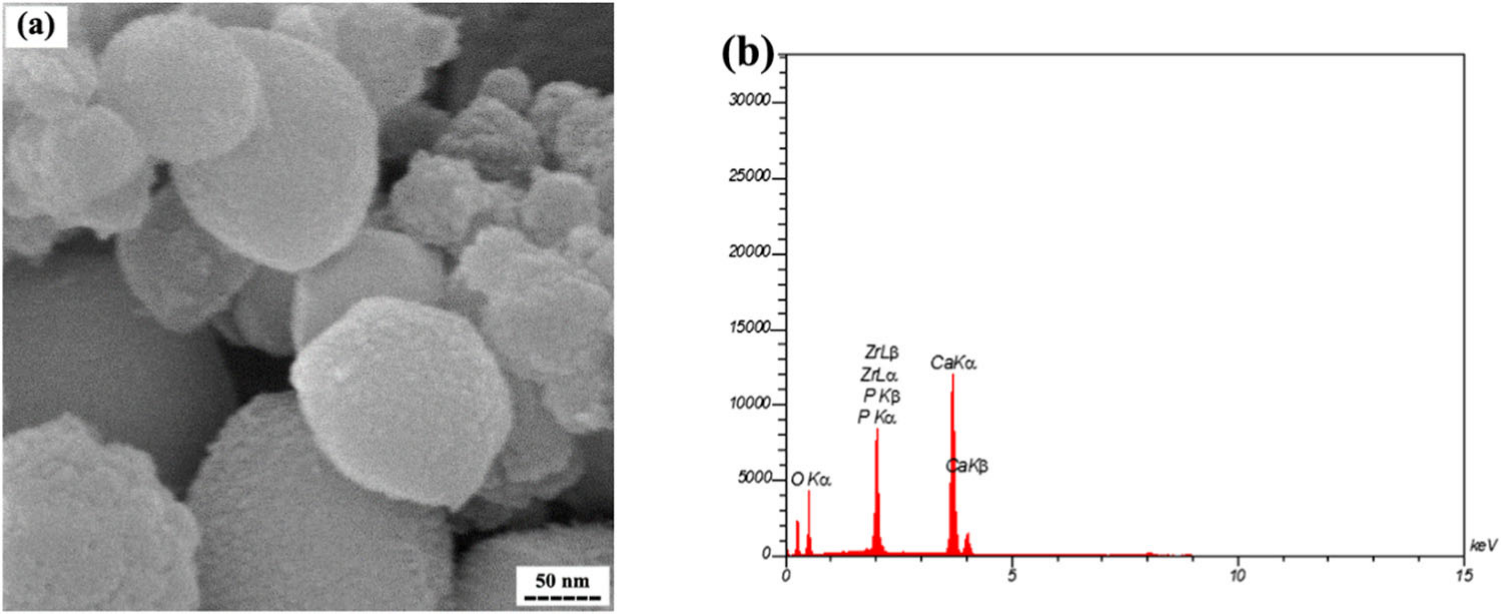
**a** SEM image, and (**b**) corresponding EDS results of synthesized powder via hydrothermal method

The morphology of the synthesized HA particles was investigated by SEM, as illustrated in Fig. 4a. The particles exhibit a predominantly spherical morphology with an average diameter of approximately 80 nm. The micrograph reveals a high degree of agglomeration in the synthesized powder. To confirm the composition of the synthesized HA nanoparticles, energy dispersive spectroscopy (EDS) was employed, as depicted in Fig. 4b. The EDS results indicate that the synthesized HA nanoparticles are primarily composed of elements Ca, P, and O. Additionally, minor peaks in the EDS spectrum suggest the presence of Zr ions resulting from the ball milling process.

**Fig. 5:**
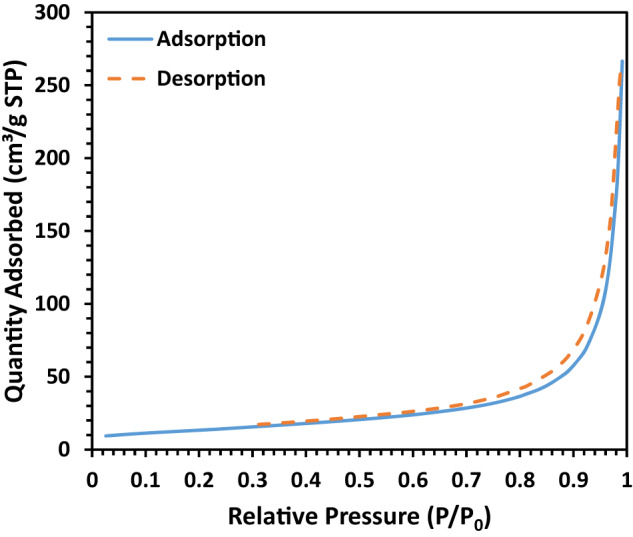
Nitrogen adsorption/desorption isotherms at ~77 K for synthesized HA powder

The BET test was utilized to determine the textural properties of the synthesized HA nanopowder. Figure 5 illustrates the corresponding N_2_ adsorption/desorption isotherms obtained through BET analysis. Research suggests that the shape and size of synthesized HA powder can be controlled by adjusting hydrothermal temperature, time, and reaction concentration [33]. The specific surface area of the material was determined to be ~48 m^2^/g, and the pore size was found to be broader in the mesoporous range, with an average diameter of ~10 nm and total pore volume of ~0.4 cm^3^/g. The increased internal porosity and specific surface area of HA are known to accelerate the healing of bone defects. Moreover, porous HA particles have been explored to maximize the porosity and surface area of osteoinductive scaffolds. Dawson et al. [34] demonstrated that a high surface area of porous HA leads to an increased rate of material resorption, resulting in physical degradation and potentially accelerating the osteoclastic breakdown of HA.

### Mechanical properties of fabricated scaffolds

**Fig. 6:**
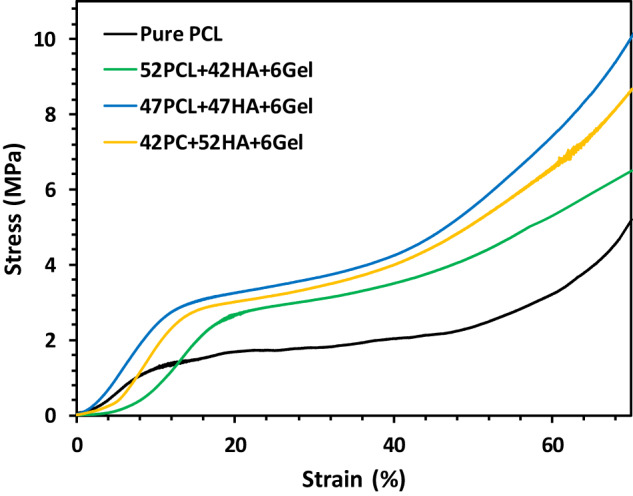
Compressive strain-stress curves of different scaffolds

The mechanical properties, a critical parameter in implant design for tissue engineering, were evaluated for four different fabricated scaffolds. The stress-strain curves and corresponding data are presented in Fig. 6 and Table 3, respectively. As strain increased, the curves deviated from linearity, revealing distinct values of strength and strain at break depending on the HA concentration. It was observed (Table 3) that increasing the HA content to 52 wt.% enhanced the compressive modulus and compressive strength of the pure PCL scaffold from 0.16 ± 0.02 to 0.31 ± 0.02 GPa and from 5.2 ± 0.2 to 9.9 ± 0.3 MPa, respectively. Similar findings have been reported in the literature [35]. The mechanical properties obtained fall within the reported range for trabecular bone (2–6 MPa compressive strength and 0.1–0.3 GPa elastic modulus) [36]. Table 3 also illustrates that the compressive strength and modulus of the nanocomposite scaffold improved with a higher HA content. This improvement is attributed to the high elastic modulus and crystallinity of HA [37, 38] compared to PCL. While pure PCL scaffolds lack the necessary mechanical and bioactive properties for bone regeneration, using a high HA content in the scaffold composition could slightly impact the mechanical properties due to discontinuities between HA particles. In other words, a higher PCL content may enhance flexibility but could impact the overall stiffness of the scaffold.

**Table 3:** Mechanical properties (*n* = 3) of scaffolds with different compositions

Scaffold composition	Compressive modulus (GPa)	Yield strength (MPa)	Compressive strength (MPa)
Pure PCL	0.16 ± 0.05	1.2 ± 0.1	5.2 ± 0.2
52PCL + 42HA+6Gel	0.25 ± 0.03	2.3 ± 0.2	6.5 ± 0.3
47PCL + 47HA+6Gel	0.29 ± 0.03	2.9 ± 0.2	9.9 ± 0.3
42PCL + 52HA+6Gel	0.31 ± 0.02	2.8 ± 0.1	8.5 ± 0.4

In conclusion, the mechanical properties of the fabricated scaffolds, particularly in the optimal composition, exhibit characteristics that make them suitable for bone cancer treatment. While the compressive modulus is slightly higher than that of natural trabecular bone, the compressive strength and yield strength fall within or above the reported ranges for trabecular bone. These scaffolds have the potential to provide the necessary mechanical support for bone cancer patients undergoing treatment, emphasizing their utility in load-bearing applications for bone regeneration and functional recovery. Therefore, the scaffold with the optimal composition of 47% PCL + 47% HA + 6% Gel exhibited the best mechanical properties and was selected for further investigation in this study.

### Biodegradability of the HA/PCL/Gel scaffold

**Fig. 7:**
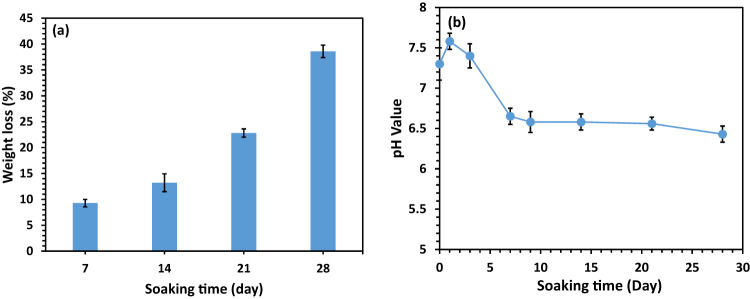
**a** Mass variation of the scaffold, and (**b**) changes in the pH value of the PBS solution during the soaking of the 47%PCL + 47%HA + 6%Gel scaffold for 28 days (*n* = 3, error bars are standard deviation)

Figure 7a illustrates the weight loss graph of the scaffold with a composition of 47% PCL, 47% HA, and 6% Gel during immersion in the PBS solution from 7 to 28 days. The graph indicates low biodegradability until 14 days, with a significant increase in weight loss after 21 days. The maximum weight loss, approximately 40%, was observed in the HA/PCL/Gel scaffold after 28 days. Given the minimal degradation of PCL over 28 days, the weight loss in the scaffold primarily results from the decomposition of HA nanoparticles and gelatin. These observations align with a previous study that highlighted the higher hydrophilicity of HA/PCL scaffolds, attributed to hydrophilic HA nanoparticles, allowing water infiltration and slightly faster degradation of HA/PCL scaffolds [39].

In Fig. 7b, the pH of the PBS solution is documented during the soaking of various samples. Initially, the pH of the medium experiences a slight increase from the original level (~7.4) to approximately 7.6. This initial rise is attributed to the degradation of gelatin, causing an increase in pH during the first three days of incubation. The presence of PCL in the scaffold contributes to a subsequent decrease in the pH of the medium during degradation, likely due to the acidic degradation product of PCL, typically the carboxyl end groups. Other researchers [40, 41] have noted that the presence of bioactive particles can compensate for the acidification of PBS caused by acidic polymer degradation products. However, HA exhibits a reverse effect, potentially accelerating the rate of degradation.

### In vitro bioactivity assessment of the HA/PCL/Gel scaffold

**Fig. 8:**
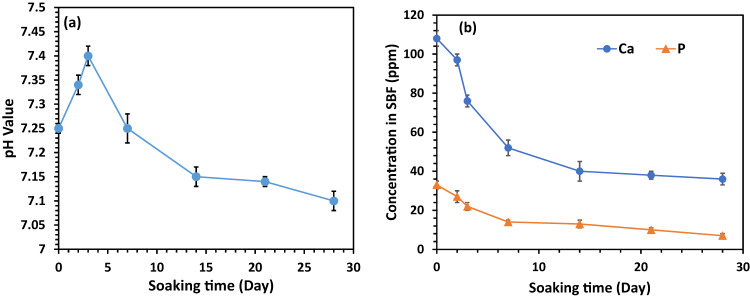
Changes of (**a**) pH value, and (**b**) Ca, and P ions of the SBF solution during the soaking of 47%PCL + 47%HA + 6%Gel scaffold for 28 days (*n* = 3, error bars are standard deviation)

In Fig. 8a, the pH values of the SBF solution change the soaking of samples from the 47% PCL + 47% HA + 6% Gel scaffold for different periods. Over the initial three days, there was an increase in the pH of the SBF solution, attributed to the release of OH^-^ ions from calcium hydroxide in the HA nanoparticles. This rise in pH aligns with observations from previous studies on the immersion of HA samples in SBF [42, 43]. Subsequently, the pH gradually decreases, likely due to the consumption of OH^-^ during apatite deposition. An ICP test was conducted to analyze the concentrations of Ca and P ions in the SBF solution after 28 days of soaking the scaffold (Fig. 8b). The concentrations of Ca and P ions showed a reduction after three days, indicating a higher rate of apatite formation, particularly on the surface of the scaffold. This in vitro bioactivity analysis aligns with previous research, emphasizing that the presence of HA promotes the production of crystalline apatite layers in SBF, showcasing its remarkable bioactivity [44, 45].

**Fig. 9:**
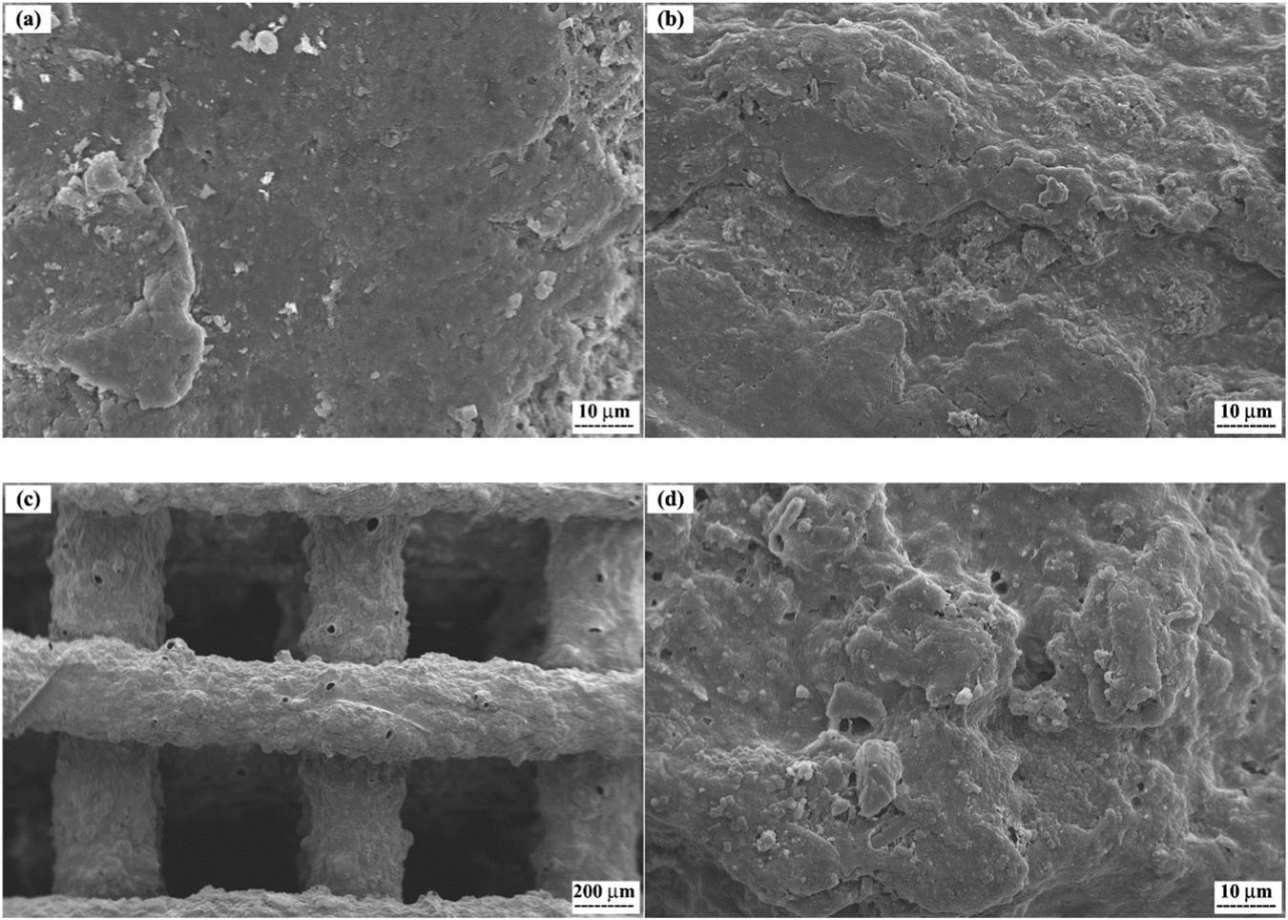
SEM micrographs showing the surface of the scaffolds with the composition of 47%PCL + 47%HA + 6%Gel, (**a**) as fabricated, (**b**) after immerging in SBF for 14 days, and (**c**, **d**) after immerging in SBF for 28 days at two different magnifications

In Fig. 9, SEM was employed to examine the surface morphology of the 47% PCL + 47% HA + 6% Gel scaffold before and after soaking in the SBF solution. The immersion of bioactive compounds in SBF is a well-established method to assess in vitro apatite formation potential. However, the reliability of this method depends on the type of bioceramics tested. Studies have indicated that carbonate-based bioceramics may not exhibit obvious apatite formation when soaked in SBF for a short time [46]. Contrary to this, the current results reveal the formation of an apatite layer on the scaffold’s surface after 14 days of soaking (Fig. 9b). Theoretically, a higher HA content enhances bioactivity, and the presence of even trace amounts of Zr ions influences this bioactivity. This observation aligns with findings by Montazerian et al. [47], who reported that the presence of Zr ions improves the time required for HA formation. Notably, after 28 days of soaking, a thick layer of fine particles developed on the scaffold’s surface (Fig. 9c, d). The formation of an apatite layer can be attributed to the presence of negatively charged OH^-^ and PO_4_^3-^ ion groups in the HA structure. These groups attract positive Ca^2+^ ions from the surrounding SBF solution, leading to a positively charged surface. This enhances the attraction of negatively charged OH^-^ and PO_4_^3^^−^ ions from the surrounding SBF solution, initiating the formation of an HA layer on the scaffold surface. This repeated reaction over time results in a well-developed bioactive surface. The SEM results, indicative of the coated samples, affirm their bioactive properties based on their chemical behavior [48, 49].

### In vitro investigation of DOX release from drug-loaded HA/PCL/Gel scaffold

**Fig. 10:**
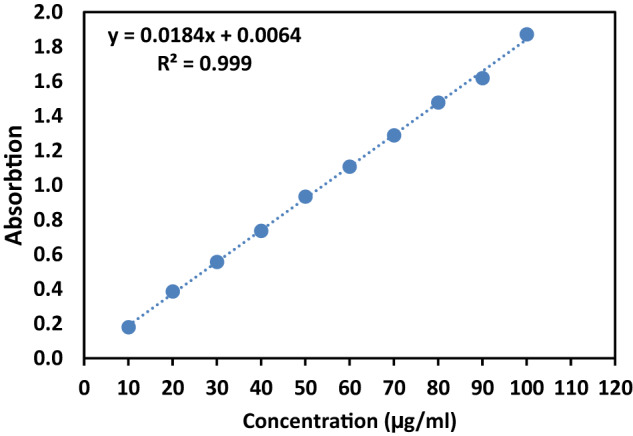
Calibration curve for DOX

In Section, 'Drug loading and releasing', the calibration curve for DOX in PBS was established, and the resulting curve is depicted in Fig. 10. The regression equation for the standard addition curve was determined as follows: y = 0.0184x + 0.0064, where y represents the absorbance value, and x is the DOX concentration. The high R^2^ value of 0.999 indicates excellent linearity for the equation, demonstrating its reliability for determining DOX concentrations within the range of 10–100 μg/ml.

**Fig. 11:**
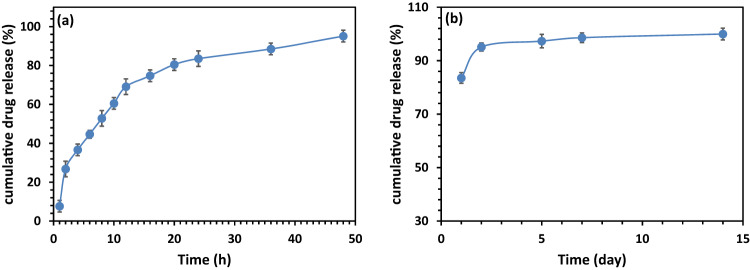
**a**, **b** Drug release profile for DOX from 47%PCL + 47%HA + 6%Gel scaffold (*n* = 3, error bars are standard deviation)

Before investigating the DOX release behavior from the 47%PCL + 47%HA + 6%Gel scaffold, LC and EE were calculated. DOX exhibited an EE of 30.8 ± 5.6%. For experimental purposes, the LC of the drug was set at 1.5%. UV-Vis spectrophotometry was then employed to determine the concentration of DOX released from the scaffold in the PBS solution for 14 days. Figure 11 illustrates the cumulative release of DOX from the fabricated nanocomposite scaffold with the 47%PCL + 47%HA + 6%Gel composition. The release process was characterized by two distinct stages: burst and sustained release. The burst release extended until day 5, after which the drug release rate stabilized. UV-Vis spectroscopy results indicated that the sample released the maximum amount of DOX (94%), with an exceptionally high release rate during the initial two days. This initial burst release was attributed to the rapid desorption of the drug from the scaffold surfaces. By day 14, the amount of released drug gradually decreased at a relatively constant rate. The calculated amounts of drug released from the nanocomposite scaffold in the PBS solution were influenced by the scaffold composition. In this study, a moderate amount of HA (47%) acted as a barrier against the release of the drug from the nanocomposite scaffolds during the first two days. Previous studies have indicated that samples with higher HA content tend to have higher encapsulation efficiency, suggesting that increasing the HA content in the scaffold could decrease the drug release rate [50]. Advanced methods for drug integration into scaffolds, including precise 3D printing of drug patterns, have been recently employed. These technologies offer more precise control over the location and dosage of drugs, their proximity to target sites, and their distance from cells, leading to more efficient drug loading and release [51]. It should be noted that the achievement of a stable drug release behavior for 14 days in the fabricated scaffolds in the current study holds significant implications for their effectiveness in bone tumor treatment. The stable drug release for 14 days implies a prolonged and sustained exposure of the tumor site to the therapeutic agent, in this case, DOX. This extended duration is crucial for maximizing the therapeutic effect on cancer cells over an extended period. In other words, sustaining drug release for 14 days helps maintain an optimal concentration of the anticancer drug within the local microenvironment. Consistent drug levels are essential for ensuring that cancer cells are continuously exposed to effective concentrations, minimizing the risk of drug resistance and enhancing treatment outcomes. Also, localized and sustained drug release at the tumor site is advantageous for minimizing systemic side effects. By reducing systemic exposure to the drug, the potential for adverse effects on healthy tissues and organs is mitigated, improving the overall safety profile of the treatment.

### In vitro biological assay

**Fig. 12:**
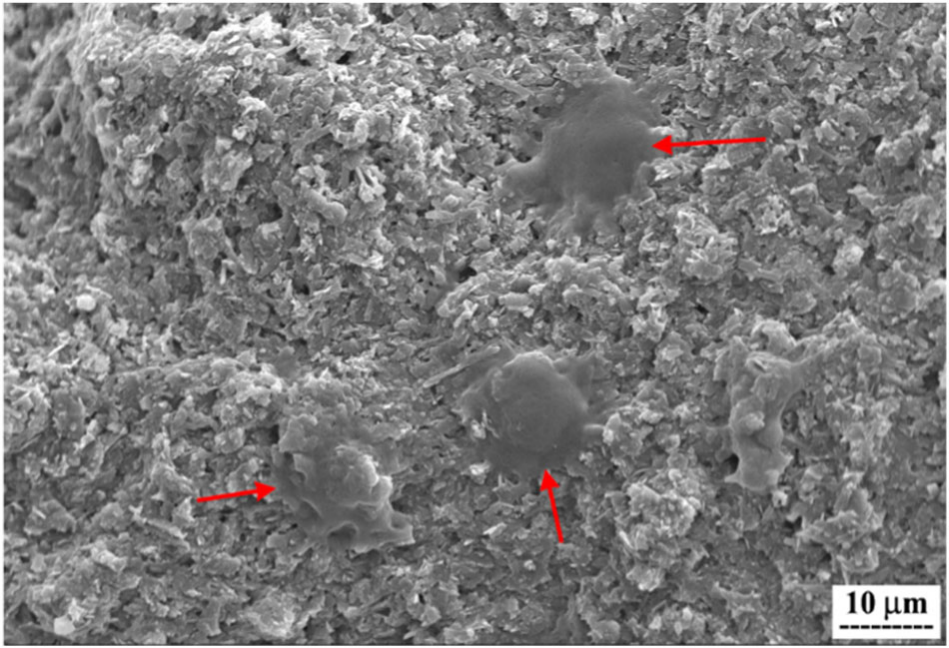
SEM micrograph showing adhesion of MG-63 cells on the surface of the 47%PCL + 47%HA + 6%Gel scaffold after 24 h. The attached cells were indicated with red arrows

The ability of cells to adhere to the scaffold’s surface is a key indicator of its biocompatibility. Cell adhesion is essential for the initial stages of tissue integration and regeneration. In the context of bone cancer treatment, a scaffold that promotes cell adhesion is advantageous. It supports the attachment of both healthy and cancerous cells, facilitating their interaction with the scaffold and influencing subsequent cellular behaviors. For this reason, the morphology and attachment capability of MG-63 cells were studied after one day of culture on the 47%PCL + 47%HA + 6%Gel scaffold. As depicted in Fig. 12, there was a favorable interaction between MG-63 cells and the scaffold, with well-spread cells exhibiting a relatively rounded morphology and lower cytoplasmic spreading compared to normal cells. The presence of Gel is thought to have enhanced cell attachment and spreading. Some studies suggest that the arginine-glycine-aspartic acid (RGD) sequence in Gel plays a significant role in establishing stable interactions between cells and the surrounding extracellular matrix, making it highly effective for cell adhesion in clinical applications [52]. The incorporation of Gel into the composite resulted in the development of a suitable scaffold with optimal bioactivity, biocompatibility, and mechanical properties.

**Fig. 13:**
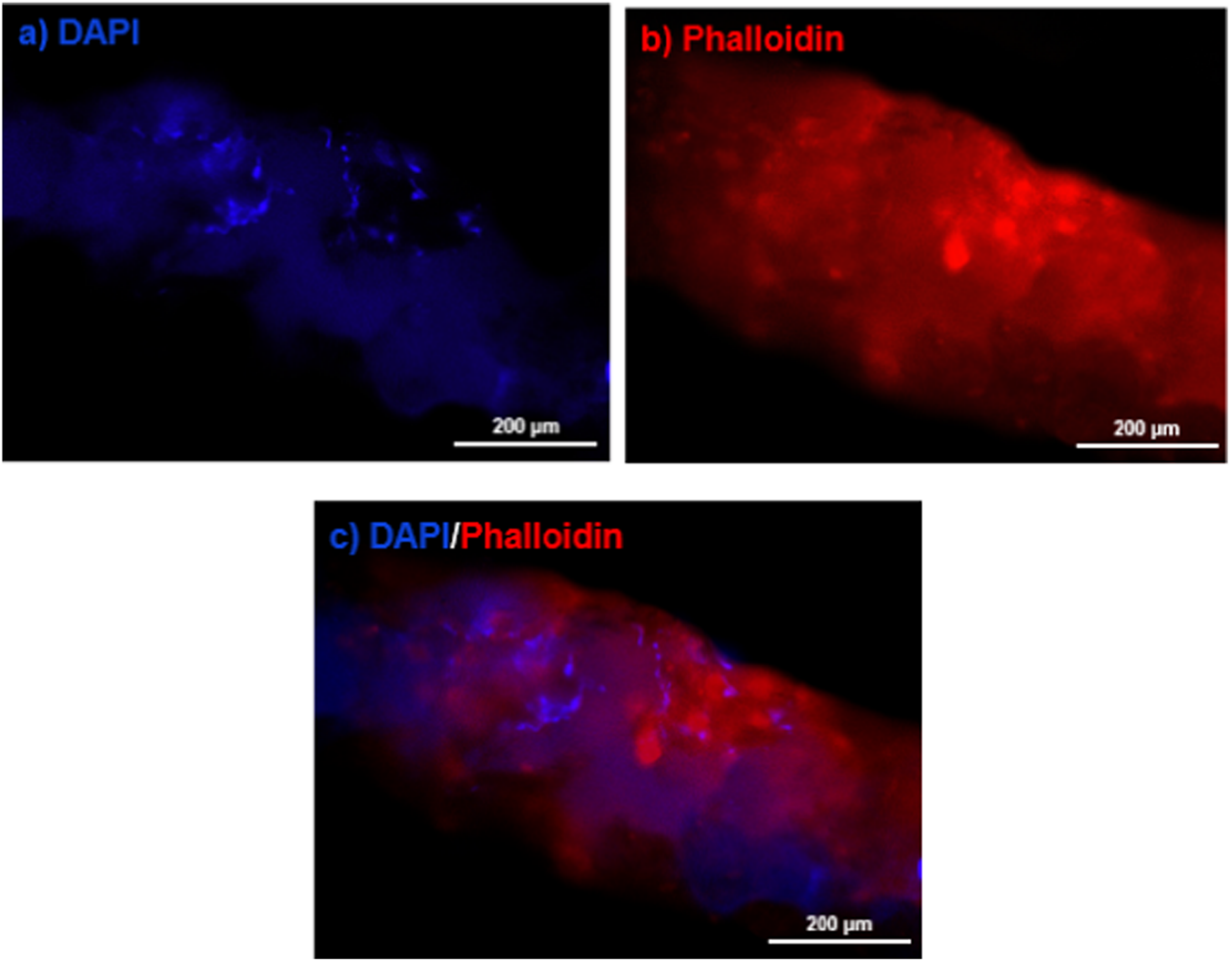
Fluorescence staining of (**a**) the nuclei (blue), (**b**) cytoskeleton (red), and (**c**) merged image of (**a**, **b**), showing MG-63 cells on the surface of the 47%PCL + 47%HA + 6%Gel scaffold after 24 h

The cell nuclei were stained with DAPI to observe the density of cell nuclei on the 47%PCL + 47%HA + 6%Gel scaffold (Fig. 13a). Additionally, filamentous actins were stained with phalloidin to analyze the cytoskeletal features of the cells (Fig. 13b) [53, 54]. On the first day, DAPI staining revealed an acceptable number of cells adhered to the scaffold, with a relatively high density of cells on the strands of the printed scaffold. Simultaneously, the relatively low cytoplasmic expansion in the images obtained from phalloidin staining indicated that the cells did not exhibit significant spreading after one day. Overall, the results of this staining confirmed the reasonable attachment and morphology of the cells on the scaffold.

**Fig. 14:**
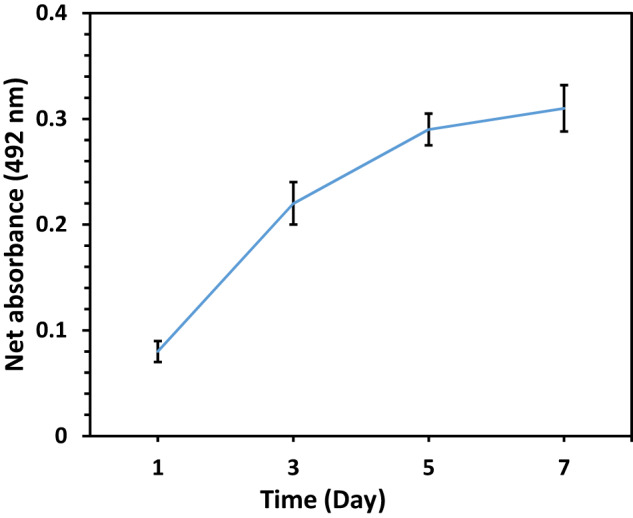
MTS results (cell metabolic activity) for MG-63 cells attached on the 47%PCL + 47%HA + 6%Gel scaffold in seven days (*n* = 3, error bars are standard deviation)

Cytotoxicity assessments provide insights into how the fabricated scaffold interacts with cells. A lack of cytotoxic effects indicates that the scaffold components do not induce harmful responses in cells, ensuring a biocompatible environment. The absence of cytotoxicity is particularly important for cancer treatment, as it ensures that the scaffold itself does not compromise the viability of normal cells in the surrounding tissue. This is critical for maintaining overall tissue health and facilitating the integration of the scaffold with the host environment. To do this assessment, the MTS test was employed to determine the viability and activity of MG-63 cells seeded on the 47%PCL + 47%HA + 6%Gel scaffold after 1, 3, 5, and 7 days of culture (Fig. 14). The MTS test confirmed the sensible attachment of MG-63 cells on the surface of the scaffold on the first day. Furthermore, the increasing trend until the seventh day indicated a significant rise in the vital capacity of the cells over the study period, which can be attributed to their proliferation and expansion on the strands. The growth curve appeared as a logarithmic pattern that reached the stationary phase on the seventh day. Generally, the exponential growth curve of the cells in this test approved the non-toxicity of the biological materials used in scaffold preparation, as well as the scaffold’s non-destructive effect on cell activity. Chuenjitkuntaworn et al. [55] illustrated that a 3D PCL/HA scaffold can support cell growth and osteogenic differentiation, implying that a 3D porous PCL/HA scaffold could be a potential candidate material for bone tissue engineering.

## Discussion

The results obtained from the XRF, XRD, and SEM analyses provided a comprehensive understanding of the characteristics of Acropora coral. The presence of calcium carbonate (CaCO_3_) as the major component and the identification of trace elements align with previous studies [28]. Similarly, the synthesized HA powder exhibited a Ca/P molar ratio close to the theoretical value of HA [31], confirming the success of the hydrothermal synthesis. The SEM images illustrated the spherical morphology of the nanoparticles, a crucial factor for their application in bone tissue engineering [33].

The mechanical properties of the fabricated scaffolds, crucial for their implantation in tissue engineering, were systematically evaluated. The compressive modulus and strength increased with higher HA content, aligning with previous studies [35]. However, it’s noteworthy that an excessively high HA content may introduce discontinuities, affecting mechanical properties. The 47% PCL + 47% HA + 6% Gel composition demonstrated optimal mechanical properties, making it a suitable candidate for further investigations.

The biodegradability assessment revealed a gradual weight loss, primarily attributed to the decomposition of HA nanoparticles and gelatin. The influence of PCL degradation on the pH of the medium was observed, emphasizing the complex interplay of scaffold components during degradation [40, 41]. The in vitro bioactivity evaluation demonstrated the scaffold’s ability to induce apatite formation, a promising sign for its performance in bone tissue engineering [42, 43].

The drug release profile of DOX from the nanocomposite scaffold exhibited a biphasic pattern, with an initial burst release followed by sustained release. The composition of the scaffold significantly influenced the release rate, with higher HA content acting as a barrier during the initial release. This finding is consistent with previous studies, emphasizing the importance of scaffold composition in drug delivery systems [50].

The in vitro biological assay confirmed the biocompatibility of the scaffold. Cell attachment, morphology, and metabolic activity of MG-63 cells on the scaffold indicated its potential for supporting cell growth and proliferation. The presence of Gel likely contributed to enhanced cell attachment, emphasizing the role of scaffold composition in cellular interactions [52].

The observed results align with existing literature on scaffold development for bone tissue engineering. The mechanical properties of the fabricated scaffold fall within the reported range for trabecular bone, emphasizing their suitability for load-bearing applications [36]. The gradual degradation of the scaffold and its ability to induce apatite formation corroborate findings from similar studies, underlining the potential for effective integration with the host tissue [39, 42, 43].

The synthesized scaffold, with its optimal mechanical properties, controlled biodegradability, and demonstrated in vitro bioactivity, holds significant promise for bone tissue engineering applications. The ability to finely tune the scaffold composition, influencing both mechanical and drug release properties, opens avenues for personalized approaches in regenerative medicine. The sustained release of DOX from the scaffold suggests its potential for localized drug delivery in cancer therapy, enhancing the therapeutic efficacy while minimizing systemic side effects.

The study likely identified an optimal scaffold composition (e.g., 47% PCL, 47% HA, 6% Gel) for achieving a balance between mechanical properties, biodegradability, and drug release. Further research can explore variations in composition to fine-tune these properties and potentially enhance specific aspects such as drug release kinetics or scaffold strength. The success of incorporating HA, PCL, and Gel opens avenues for integrating additional bioactive agents. Researchers can explore the inclusion of growth factors, antimicrobial agents, or other therapeutic molecules to create multifunctional scaffolds. This approach aims to address various aspects of bone cancer treatment, such as promoting tissue regeneration or preventing infections. Building on the controlled drug release observed in this study, further research can delve into advanced drug delivery strategies. This may include incorporating stimuli-responsive materials for on-demand drug release, exploring combination therapies with multiple drugs, or utilizing nanotechnology for precise control over drug delivery patterns. In conclusion, applying the findings from this study to further research involves exploring new avenues for scaffold improvement, incorporating advanced technologies, and progressing toward preclinical and clinical validations. This iterative process is essential for developing innovative and effective solutions for bone cancer treatment.

While the current study provides valuable insights, it is not without limitations. The in vitro assessments, while informative, do not fully replicate the complex in vivo environment. Further studies, including in vivo experiments, are essential to validate the scaffold’s performance in a more physiological setting. Additionally, exploring alternative drug-loading techniques and investigating the long-term effects of scaffold degradation are avenues for future research. The expansion of nanocomposite scaffold production for clinical applications may face challenges and limitations such as scalability issues, reproducibility concerns, regulatory hurdles, cost implications, and potential safety and toxicity considerations. Additionally, the complexity of the manufacturing process and the need for specialized expertise could pose obstacles to widespread scalability. It is crucial to address these factors to ensure the successful and efficient translation of nanocomposite scaffolds from laboratory settings to clinical applications.

Addressing these challenges requires a comprehensive approach involving technological innovation, regulatory compliance, quality assurance, and close collaboration between researchers, manufacturers, and regulatory bodies. Despite these challenges, overcoming them can unlock the potential of nanocomposite scaffolds for impactful clinical applications in tissue engineering and regenerative medicine.

## Conclusion

In this research, HA nanoparticles were successfully synthesized from coral using a hydrothermal method, showcasing a spherical morphology with an average diameter of approximately 80 nm. The study focused on the fabrication of 3D-printed scaffolds using HA, PCL, and Gel, exhibiting porous structures with optimal mechanical properties akin to trabecular bone. These scaffolds demonstrated exceptional bioactivity, forming crystalline apatite layers, and showed a controlled drug release profile. Additionally, the presence of Gel enhanced cell attachment and spreading, making the HA/PCL/Gel scaffolds promising candidates for bone tissue engineering applications. In conclusion, while the use of Acropora coral-derived HA nanoparticles in scaffolds for bone cancer treatment holds promise, thorough investigations and optimization are necessary to ensure the safety and efficacy of these scaffolds in a clinical setting.

## Data Availability

The data presented in this study are available on request from the corresponding author.

## References

[1] Francois EL, Yaszemski MJ (2018, August 17). Preclinical Bone Repair Models in regenerative medicine. Principles of Regenerative Medicine (Third Edition). Retrieved August 9, 2018.

[2] Amini AR, Laurencin CT, Nukavarapu SP. Bone Tissue Engineering: Recent Advances and Challenges. Crit Rev Biomed Eng. 2012;40:363–408. 10.1615/critrevbiomedeng.v40.i5.10. 23339648 10.1615/critrevbiomedeng.v40.i5.10 PMC3766369

[3] Alidadi S. Nanoscale bioceramics in bone tissue engineering-an overview. Indian J Veter Sci Biotechnol. 2020;16:7–11.

[4] Feng J, Thian ES. Applications of nanobioceramics to healthcare technology. Nanotechnol Rev. 2013 10.1515/ntrev-2012-0065.

[5] Singh Z. Nanoceramics in Bone Tissue Engineering: The Future Lies Ahead. Trends J Sci Res. 2018;3:120 10.31586/nanomaterials.0303.03.

[6] Ma H, Feng C, Chang J, Wu C. 3D-printed bioceramic scaffolds: From bone tissue engineering to tumor therapy. Acta Biomaterialia. 2018;79:37–59. 30165201 10.1016/j.actbio.2018.08.026

[7] Du X, Fu S, Zhu Y. 3D printing of ceramic-based scaffolds for bone tissue engineering: an overview. J Mater Chem B. 2018;6:4397–412. 10.1039/c8tb00677f. 32254656 10.1039/c8tb00677f

[8] Wang C, Huang W, Zhou Y, He L, He Z, Chen Z, et al. 3D printing of bone tissue engineering scaffolds. Bioactive Mater. 2020;5:82–91. 10.1016/j.bioactmat.2020.01.004. 10.1016/j.bioactmat.2020.01.004 PMC6962643 31956737

[9] Salgado AJ, Coutinho OP, Reis RL. Bone Tissue Engineering: State of the Art and Future Trends. Macromol Biosci. 2004;4:743–65. 10.1002/mabi.200400026. 15468269 10.1002/mabi.200400026

[10] Fiume E, Magnaterra G, Rahdar A, Verné E, Baino F. Hydroxyapatite for Biomedical Applications: A Short Overview. Ceramics. 2021;4:542–63. 10.3390/ceramics4040039.

[11] Marew T, Birhanu G. Three dimensional printed nanostructure biomaterials for bone tissue engineering. Regen Ther. 2021;18:102–11. 10.1016/j.reth.2021.05.001. 34141834 10.1016/j.reth.2021.05.001 PMC8178073

[12] Wen Y, Xun S, Haoye M, Baichuan S, Peng C, Xue-Jian L, et al. 3D printed porous ceramic scaffolds for bone tissue engineering: a review. Biomaterials Sci. 2017;5:1690–8. 10.1039/c7bm00315c. 10.1039/c7bm00315c 28686244

[13] Zhang L, Yang G, Johnson BW, Jia X. Three-dimensional (3D) printed scaffold and material selection for bone repair. Acta Biomaterialia. 2019;84:16–33. 10.1016/j.actbio.2018.11.039. 30481607 10.1016/j.actbio.2018.11.039

[14] Venkatesan J, Kim S. Nano-Hydroxyapatite Composite Biomaterials for Bone Tissue Engineering—A Review. J Biomed Nanotechnol. 2014;10:3124–40. 10.1166/jbn.2014.1893. 25992432 10.1166/jbn.2014.1893

[15] Zheng Y, Liu X, Yeung KW, Liu C, Yang X. Biomimetic porous scaffolds for bone tissue engineering. Mater Sci Eng R. 2014;80:1–36. 10.1016/j.mser.2014.04.001.

[16] Vincent JFV. Biomimetics — a review. Proc Inst Mech Eng, Part H: J Eng Med. 2009;223:919–39. 10.1243/09544119jeim561. 10.1243/09544119JEIM561 20092091

[17] Hu J, Russell JJ, Ben-Nissan B, et al. Production and analysis of hydroxyapatite from Australian corals via hydrothermal process. J Mater Sci Lett. 2001;20:85–87.

[18] Green DW, Ben-Nissan B, Yoon KS, Milthorpe B, Jung HS. Natural and Synthetic Coral Biomineralization for Human Bone Revitalization. Trends Biotechnol. 2017;35:43–54. 27889241 10.1016/j.tibtech.2016.10.003

[19] Demers CN, Hamdy C, Corsi K, Chellat F, Tabrizian M, Yahia L. Natural coral exoskeleton as a bone graft substitute: a review. Bio-Med Mater Eng. 2002;12:15–35. 11847406

[20] Manassero M, Viateau V, Deschepper M, Oudina K, Logeart-Avramoglou D, Petite H, et al. Bone Regeneration in Sheep Using AcroporaCoral, a Natural Resorbable Scaffold, and Autologous Mesenchymal Stem Cells. Tissue Eng Part A. 2013;19:1554–63. 10.1089/ten.tea.2012.0008. 23427828 10.1089/ten.TEA.2012.0008

[21] Hamlekhan A, Mozafari M, Nezafati N, Azami M, Hadipour H. A proposed fabrication method of novel PCL-GEL-HA nanocomposite scaffolds for bone tissue engineering applications. Adv Compos Lett. 2010;19:4.

[22] Shi H, Zhou Z, Li W, Fan Y, Li Z, Wei J. Hydroxyapatite based materials for bone tissue engineering: A brief and comprehensive introduction. Crystals. 2021;11:149.

[23] Gómez-Lizárraga KK, Flores-Morales C, Del Prado-Audelo ML, Alvarez-Pérez MA, Pina-Barba MC, Escobedo C. Polycaprolactone- and polycaprolactone/ ceramic-based 3D-bioplotted porous scaffolds for bone regeneration: a comparative study. Mater. Sci. Eng. C. 2017;79:326–35. 10.1016/J.MSEC.2017.05.003. 10.1016/j.msec.2017.05.003 28629025

[24] Zhang K, Zhou Y, Xiao C, Zhao W, Wu H, Tang J, Li Z, Yu S, Li X, Min L, Yu Z. Application of hydroxyapatite nanoparticles in tumor-associated bone segmental defect. Sci. Adv. 2019;5:eaax6946. 31414050 10.1126/sciadv.aax6946 PMC6677551

[25] Tanner K. Bioactive composites for bone tissue engineering. Proc Inst Mech Eng, Part H: J Eng Med. 2010;224:1359–72. 10.1243/09544119jeim823. 10.1243/09544119JEIM823 21287825

[26] Bose S, Koski C, Vu AA. Additive manufacturing of natural biopolymers and composites for bone tissue engineering. Mater Horizons. 2020;7:2011–27. 10.1039/d0mh00277a.

[27] Roy DM, Linnehan SK. Hydroxyapatite formed from Coral Skeletal Carbonate by Hydrothermal Exchange. Nature. 1974;247:220–2. 4149289 10.1038/247220a0

[28] Guillemin N, Manassero M, Decambron A, Petite H, Bizios R, Viateau V. Coral Scaffolds in Bone Tissue Engineering and Bone Regeneration. In: Goffredo S, Dubinsky Z, (eds). The Cnidaria, Past, Present and Future. Cham: Springer; 2016.

[29] Farfan GA, Apprill A, Cohen A, DeCarlo TM, Post JE, Waller RG, et al. Crystallographic and chemical signatures in coral skeletal aragonite. Coral Reefs. 2021;41:19–34. 10.1007/s00338-021-02198-4.

[30] Wu YC, Lee TM, Chiu KH, Shaw SY, Yang CY. A comparative study of the physical and mechanical properties of three natural corals based on the criteria for bone-tissue engineering scaffolds. J Mater Sci Mater Med. 2009;20:1273–80. 19267261 10.1007/s10856-009-3695-3

[31] Kongsri S, Janpradit K, Buapa K, et al. Nanocrystalline hydroxyapatite from fish scale waste: preparation, characterization and application for selenium adsorption in aqueous solution. Chem Eng J. 2013;215–216:522–32.

[32] Sivakumar M, Kumar TS, Shantha K, Rao KP. Development of hydroxyapatite derived from Indian coral. Biomaterials. 1996;17:1709–14. 10.1016/0142-9612(96)87651-4. 8866033 10.1016/0142-9612(96)87651-4

[33] Ma G. Three common preparation methods of hydroxyapatite. IOP Conference Series: Mater Sci Eng. 2019;688:033057 10.1088/1757-899x/688/3/033057.

[34] Dawson E, Suzuki R, Samano M, Murphy M. Increased internal porosity and surface area of hydroxyapatite accelerates healing and compensates for low bone marrow mesenchymal stem cell concentrations in critically-sized bone defects. Appl Sci. 2018;8:1366 10.3390/app8081366.

[35] Saber-Samandari S, Saber-Samandari S. Biocompatible nanocomposite scaffolds based on copolymer-grafted chitosan for bone tissue engineering with drug delivery capability. Mater Sci Eng: C. 2017;75:721–32. 10.1016/j.msec.2017.02.112. 10.1016/j.msec.2017.02.112 28415522

[36] Lee S, Porter M, Wasko S, Lau G, Chen PY, Novitskaya EE, et al. Potential bone replacement materials prepared by two methods. Mater Res Soc Symp Proc. 2012;1418:177–88.

[37] Rezwan K, Chen QZ, Blaker JJ, Boccaccini AR. Biodegradable and bioactive porous polymer/inorganic composite scaffolds for bone tissue engineering. Biomaterials. 2006;27:3413–31. 16504284 10.1016/j.biomaterials.2006.01.039

[38] Ingole VH, Ghule SS, Vuherer T, Kokol V, Ghule AV. Mechanical Properties of Differently Nanostructured and High-Pressure Compressed Hydroxyapatite-Based Materials for Bone Tissue Regeneration. Minerals. 2021;11:1390 10.3390/min11121390.

[39] Díaz E, Sandonis I, Valle MB. In vitro degradation of poly(caprolactone)/nha composites. J Nanomater. 2014;2014:1–8. 10.1155/2014/802435.

[40] Casarin SA, Malmonge SM, Kobayashi M, Marcondes Agnelli JA. Study on in vitro degradation of bioabsorbable polymers poly(hydroxybutyrate-co-valerate)-(PHBV) and poly(caprolactone)-(PCL). J Biomater Nanobiotechnol. 2011;2:207.

[41] Bergsma JE, de Bruijn WC, Rozema FR, Bos RRM, Boering G. Late degradation tissue response to poly(L-lactide) bone plates and screws. Biomaterials. 1995;16:25–31. 7718688 10.1016/0142-9612(95)91092-d

[42] Govindan R, Girija EK. Drug loaded phosphate glass/hydroxyapatite nanocomposite for orthopedic applications. J Mater Chem B. 2014;2:5468–77. 10.1039/c4tb00549j. 32261767 10.1039/c4tb00549j

[43] Vallet-Regí M, Pérez-Pariente J, Izquierdo-Barba I, Salinas AJ. Compositional Variations in the Calcium Phosphate Layer Growth on Gel Glasses Soaked in a Simulated Body Fluid. Chem Mater. 2000;12:3770–5. 10.1021/cm001068g.

[44] Fu X, Liu P, Zhao D, Yuan B, Xiao Z, Zhou Y, et al. Effects of Nanotopography Regulation and Silicon Doping on Angiogenic and Osteogenic Activities of Hydroxyapatite Coating on Titanium Implant. Int J Nanomed. 2020;15:4171–89. 10.2147/IJN.S252936 PMC7297339 32606671

[45] Thian ES, Huang J, Best SM, Barber ZH, Bonfield W. Magnetron co-sputtered silicon-containing hydroxyapatite thin films–an in vitro study. Biomaterials. 2005;26:2947–56. 15603789 10.1016/j.biomaterials.2004.07.058

[46] Kokubo T, Takadama H. In vitro evaluation of bone bioactivity. Biocer Clin Appl. 2008 10.1201/9781439832530.ch7.

[47] Montazerian M, Yekta B, Marghussian V, Bellani C, Siqueira R, Zanotto E. Bioactivity and Cell proliferation in radiopaque gel-derived CaO–P2O5–SiO2–ZrO2 glass and glass–ceramic powders. Mater Sci Eng.: C. 2015;55:436–47. 10.1016/j.msec.2015.05.065 26117775

[48] Chavan PN, Bahir MM, Mene RU, Mahabole MP, Khairnar RS. Study of Nanobiomaterial Hydroxyapatite in Simulated Body Fluid: Formation and Growth of Apatite. Mater Sci Eng B. 2010;168:224–30.

[49] Fathi M, Hanifi A, Mortazavi V. Preparation and bioactivity evaluation of bone-like hydroxyapatite nanopowder. J Mater Proc Technol. 2008;202:536–42. 10.1016/j.jmatprotec.2007.10.004.

[50] Moghanian A, Zohourfazeli M, Tajer MH. The effect of zirconium content on in vitro bioactivity, biological behavior and antibacterial activity of sol-gel derived 58s bioactive glass. J Non-Crystalline Solids. 2020;546:120262 10.1016/j.jnoncrysol.2020.120262.

[51] Costa PA. Bone tissue engineering drug delivery. Curr Mol Biol Rep. 2015;1:87–93. 10.1007/s40610-015-0016-0. 10.1007/s40610-015-0022-2 PMC4654432 26618105

[52] Wu S-C, Chang W-H, Dong G-C, Chen K-Y, Chen Y-S, Yao C-H. Cell adhesion and proliferation enhancement by gelatin nanofiber scaffolds. J Bioactive Compatible Polymers. 2011;26:565–77. 10.1177/0883911511423563.

[53] Šimoliūnas E, Kantakevičius P, Kalvaitytė M, Bagdzevičiūtė L, Alksnė M, Baltriukienė D. DNA-dapi interaction-based method for cell proliferation rate evaluation in 3D structures. Curr Issues Mol Biol. 2021;43:251–63. 10.3390/cimb43010021. 34070775 10.3390/cimb43010021 PMC8929038

[54] Ng KW, Leong DT, Hutmacher DW. The challenge to measure cell proliferation in two and three dimensions. Tissue Eng. 2005;11:182–91. 10.1089/ten.2005.11.182. 15738673 10.1089/ten.2005.11.182

[55] Chuenjitkuntaworn B, Osathanon T, Nowwarote N, Supaphol P, Pavasant P. The efficacy of polycaprolactone/hydroxyapatite scaffold in combination with mesenchymal stem cells for bone tissue engineering. J Biomed Mater Res Part A. 2015;104:264–71. 10.1002/jbm.a.35558. 10.1002/jbm.a.35558 26362586

